# Ultra-rapid elimination of biofilms via the combustion of a nanoenergetic coating

**DOI:** 10.1186/1472-6750-13-30

**Published:** 2013-03-27

**Authors:** Byung-Doo Lee, Rajagopalan Thiruvengadathan, Sachidevi Puttaswamy, Brandon M Smith, Keshab Gangopadhyay, Shubhra Gangopadhyay, Shramik Sengupta

**Affiliations:** 1 Department of Biological Engineering, University of Missouri, 1406 E Rollins St., 252 Ag Engineering Building, Columbia, MO 65211-5200, USA; 2 Department of Electrical and Computer Engineering, University of Missouri, 349, Engineering Building West, Columbia, MO 65211, USA; 3 NEMS/MEMS Works LLC, 8850 Westlake Road West, Columbia, MO 65202, USA

**Keywords:** Nanoenergetic materials, Nanothermites, Biofilms, Biofilm removal, Nanomaterial combustion, Ultra-rapid decontamination

## Abstract

**Background:**

Biofilms occur on a wide variety of surfaces including metals, ceramics, glass etc. and often leads to accumulation of large number of various microorganisms on the surfaces. This biofilm growth is highly undesirable in most cases as biofilms can cause degradation of the instruments and its performance along with contamination of the samples being processed in those systems. The current “offline” biofilm removal methods are effective but labor intensive and generates waste streams that are toxic to be directly disposed. We present here a novel process that uses nano-energetic materials to eliminate biofilms in < 1 second. The process involves spray-coating a thin layer of nano-energetic material on top of the biofilm, allowing it to dry, and igniting the dried coating to incinerate the biofilm.

**Results:**

The nanoenergetic material is a mixture of aluminum (Al) nanoparticles dispersed in a THV-220A (fluoropolymer oxidizer) matrix. Upon ignition, the Al nanoparticles react with THV-220A exothermically, producing high temperatures (>2500 K) for an extremely brief period (~100 ms) that destroys the biofilm underneath. However, since the total amount of heat produced is low (~0.1 kJ/cm^2^), the underlying surface remains undamaged. Surfaces with biofilms of *Pseudomonas aeruginosa* initially harboring ~ 10^7^ CFU of bacteria /cm^2^ displayed final counts of less than 5 CFU/cm^2^ after being subjected to our process. The byproducts of the process consist only of washable carbonaceous residue and gases, making this process potentially inexpensive due to low toxic-waste disposal costs.

**Conclusions:**

This novel method of biofilm removal is currently in the early stage of development. However, it has potential to be used in offline biofilm elimination as a rapid, easy and environmentally friendly method.

## Background

A biofilm is defined as a microbially derived sessile community characterized by cells that are irreversibly attached to a substratum or interface or to each other; are embedded in a matrix of extracellular polymeric substances that they have produced; and exhibit an altered phenotype with respect to growth rate and gene transcription [1]. Biofilms can occur spontaneously (without deliberate intention to grow them) on a wide variety of surfaces such as metals, plastics, glass, ceramics, wood and cement. Once established, they can accommodate a large number of bacteria per unit area of the surface. While ~10^5^ - 10^7^ CFU (Colony Forming Units) of bacteria /cm^2^ are commonly encountered, numbers as high as 10^9^ – 10^10^ CFU/cm^2^ have been reported [2,3].

Their presence may be undesirable in a variety of applications. For instance, on ship hulls, the formation of a microbial biofilm can raise the drag coefficient by as much as 29% [4], contributing to correspondingly higher fuel usage. In heat exchangers and cooling water systems, which are an integral part of a wide variety of industrial processes, a 250 micron thick layer of biofilm may reduce the effective heat transfer coefficient of a heat exchanger by as much as 50% [5]. In addition, the metabolism of bacteria in the biofilm (production of carbonic, pyruvic, citric, lactic and other acids) causes a reduction in pH at the surfaces, leading to enhanced rate of chemical corrosion [6]. This inflicts additional economic burdens such as the need for premature replacement of equipment and unscheduled downtime to clean fouled equipment [7]. In the oil and natural gas industry, bacterial biofilms cause financial losses of ~ $100 Million each year through the corrosion of pipelines and process equipment and souring of reservoirs [8]. In the paper manufacturing industry, biofilms are responsible for an estimated 10-20% of all machine downtime [9]. Thus, there are huge incentives to (a) prevent biofilm formation, and (b) to minimize their growth rate during operation of a wide variety of process equipments like tanks, transport tubing, and heat exchangers. Consequently, several approaches have been explored in the past. These approaches include the use of materials and coatings that hinder biofilm formation and growth, the continuous or pulsed addition of chemicals such as acids, oxidizers or enzymes to the process fluid, and the intermittent use of mechanical cleaning agents like scrubbing balls. Despite these efforts, it is almost impossible to completely prevent biofilms from getting established, and as a result, adversely affecting the performance of the equipment [10]. Once the performance of the equipment falls below acceptable levels, they have been taken offline for biofilm removal.

The offline removal of biofilms from process equipment is also a difficult task. The common methods adopted for the offline removal of biofilm from process equipment [10] can be broadly classified into mechanical and chemical processes. The most common mechanical processes include water/steam/sand blasting for large exposed surfaces (blasting being the process of forcibly propelling a stream of material against a surface under high pressure) and abrasive pads for smaller, more difficult to reach surfaces such as the interior of tubes. The main disadvantages of using mechanical processes are that they are labor intensive and take a long time. The latter is especially undesirable, as in many cases, the whole process remains shut for the duration during which one or more of the equipments are brought offline, resulting in losses of tens of thousands of dollars an hour. The other alternative is to use strong chemical cleaning agents like acids, alkalis, and strong biocides. Strong chemicals are often required because the biofilm’s extracellular matrix prevents milder chemicals such as antibiotics and germicides from acting on the cells embedded within it. The use of chemicals for biofilm removal has its own advantages and disadvantages. While they are usually less labor intensive, relatively faster, and can act on hard-to-reach surfaces, they are often expensive. Moreover, the use of strong chemicals can also result in the generation of waste-streams that are expensive to dispose off due to their toxicity.

Thus, there is a need for an offline biofilm-removal process for process equipment that is fast, effective, economical, and yet environmentally friendly. Table 1 lists numerous approaches (ultrasonication, electric fields, mild chemicals such as enzymes, and their combinations [11-17]) that have been employed and reported by other groups for this purpose. As can be seen, their efficacy is limited (they achieve only 1 to 3 log_10_ reductions in the number of viable bacteria per unit area) and/or take a long time (hours). In contrast, if our proposed method were employed for the same application (offline removal of biofilms from process equipment), the biofilm removal could be potentially completed faster (in minutes), and with greater efficacy (> 5 log_10_ reduction in the number of viable bacteria).

**Table 1:** Efficacy of various environmentally friendly processes used for the removal of biofilms

**Biofilm type**	**Method used for biofilm elimination**	**Log reduction in CFU count**	**Time taken**
***P. aeruginosa *and *S. aureus***	Cleaning with detergents, followed by high-pressure wash and mechanical scrubbing [11]	< 3	20 min soak detergent + 1 min wash + <1 min scrub
***P. aeruginosa *and *K. pneumoniae***	Treatment with multiple chemicals (chelating agents, hypochlorites etc.) [12]	1–3 depending on chemical used	1 hr
***Pseudomonas fluorescens***	Combination of Enzymes (proteolytic + polysaccharide-degrading enzymes) [13]	2-4	2 hrs
***P. aeruginosa, S. epidermidis, and S. aureus***	Ultrasound [15]	1.02 – 1.48	10 minutes
***E. coli *and *S. aureus***	Chelating Agents (EDTA / EGTA) and Ultrasound [14]	~ 2	Unknown soak time; 10–60s sonication
***Pseudomonas aeruginosa***	Biocides (Chemicals) + Electric Field [16]	3	12 hours
***E. coli***	High Pressure CO_2_ / N_2_ aerosols [17]	1-2	90 s
***P. aeruginosa***	Our Method (Rapid combustion of a sprayed on layer of nano-energetic materials)	> 5	~ 1 min spray; < 1 s burn

We present here a novel material and method that is able to outperform the methods listed in Table 1 in both speed and efficacy. Briefly, we use an optimized blend of Al nanoparticles (fuel) in a fluoropolymer (oxidizer) matrix that is spray-coated onto the surfaces, and which burns away extremely rapidly (< 1 sec/cm^2^), generating very high temperatures [2200–3200 K [18]] that destroys the biofilm, but leaves the underlying surface intact. The underlying surface remains unaffected because the *amount* of heat released is not very high: ~ 0.1 kJ/cm^2^, according to our estimates based on the heat of combustion of Al [19], and the known loads of Al nanoparticles in our formulation. The key to the efficacy of the process lies in the use of Al nanoparticles with average size of 80 nm and narrow size distribution. The nanometer size of Al particles not only enables the rapid release of the heat of combustion (significantly reduced mass transfer limitation) along with the generation of high temperatures, but also allows us to spread a small mass of Al (~ 10 mg) uniformly over the test areas (~20 cm^2^). The latter limits the amount of heat released, which, in turn, limits the damage done to the underlying substrate. We demonstrate the efficacy of this technique using *Pseudomonas aeruginosa* biofilms grown at moderate shear as our model biofilm. *P. aeruginosa* was chosen because it has been extensively studied [20], and to compare our technique to those of other researchers [11-13] who report the efficacy of their biofilm removal/killing methods using *P. aeruginosa* biofilms.

## Methods

### Cultivation of model biofilms on substrates of interest

We cultivated *P. aeruginosa* biofilms on a variety of substrates such as metals (steel and brass), ceramics (bathroom tiles), and glass that can be expected to withstand the high temperature generated during the burning process for a very short duration.

Above-mentioned substrates with dimensions of 1″ × 3″ served as our test coupons (except for the ceramic, for which a 2″ × 2″ piece was used instead). These substrates were first thoroughly cleaned to ensure no pre-existing biofilm. A 1% (w/v) solution of detergent was prepared, and the substrates were first cleaned by sonicating them in this solution for 10 minutes using an ultrasonic bath. The detergent solution was then replaced with DI water and the substrates were sonicated for an additional 10 minutes. The substrates were then rinsed with DI water and then placed in 2 M HCl (for glass and ceramic), or bleach solution (for metals). The substrates were then sonicated again in DI water and rinsed. Finally, they were air-dried in a Biological Safety Cabinet.

Cultures of *P. aeruginosa* were obtained from commercial sources (Ward’s Natural Sciences), and an aliquot was inoculated into 10 ml of TSB (Tryptic Soy Broth) and incubated overnight with shaking at 37°C. The resulting log-culture had a concentration of ~10^9^ CFU/ml. The bacterial cells were isolated by centrifugation, and re-suspended in an equal volume (10 ml) of 1× Phosphate Buffered Saline (PBS) (a buffer consisting of Sodium Chloride and Sodium Phosphate). This suspension of bacteria was then introduced into a sterile (autoclaved) vessel with a capacity of ~ 1 L loaded with ~500 ml of 1/10 × TSB. Multiple (four to six) coupons of a particular material (metal, glass or ceramic) were prepared by covering one side with a piece of adhesive backed silicone rubber sheet, loaded into the tank with the exposed side upwards (in contact with the liquid), the top of the tank covered in saran wrap, and the tank placed in an incubator-shaker. The incubator shaker was operated at room temperature (~ 25°C) with an oscillation speed of 200 rpm, which corresponds to a shear rate of ~10^5^ s^-1^ at the fluid-solid interface (biofilm). The biofilm was allowed to form over a period of 4 days. At the end of this period, during which there was perceptible growth of biofilm in the system, the coupons were extracted, washed in DI water to remove cells that adhere weakly to the surface (those not within the biofilm matrix) and loaded into individual Ziploc™ bags (pre-sterilized by wiping with 70% ethanol and exposed to UV radiation in a biological safety cabinet) and stored in refrigerator (4°C). They were then used (within a period of 3 days) for further testing. It may be noted that the biofilms still retain their characteristic slimy appearance after retrieval from storage, indicating that they remain in a hydrated state.

### The biofilm removal process

**Figure 1:**
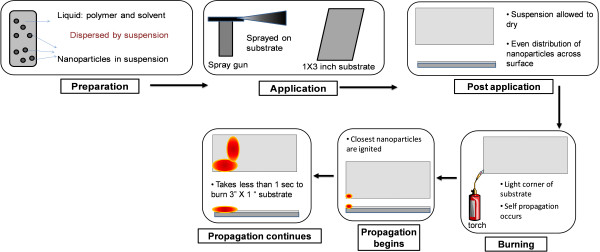
Represents the schematic of the proposed process for ultra-rapid removal of biofilms, which involves spray coating the nanoparticles in suspension onto the substrate containing biofilm, followed by burning the entire surface of the substrate by initiating the ignition of one of its corners.

Our proposed process to eliminate biofilms from substrates of interest is illustrated schematically in Figure 1. As shown in Figure 1, we begin the process by dissolving a known amount of THV 220A in acetone using sonication. THV 220A is a commercially available (3 M, St. Paul, MN) fluoropolymer, composed of tetrafluoroethylene, hexafluoropropylene and vinylidene-fluoride. Al nanoparticles are then added to this solution and dispersed homogeneously using an ultrasonic bath. The fluoropolymer plays the role of oxidizer and the Al nanoparticles plays the role of fuel in the nanoenergetic composition. The amount of acetone used in the dispersions was varied as 7, 8, and 10 ml for a total mass of 500 mg of THV 220A polymer and Al nanoparticles. The amount of Al nanoparticles and THV 220A were varied suitably so that the weight ratio of Al to THV 220A was kept at 1:9, 2:8, and 3:7. The nanoenergetic dispersion was then sprayed uniformly on top of the surfaces of interest (biofilm-covered surfaces of different materials).The acetone evaporates rapidly, leaving behind a dry, paint-like coating on the surface. In order to keep the thickness of the coating nearly the same on any given substrate, the same volume (7 ml) of fluoropolymer/nanoparticle and acetone blend was uniformly used, yielding nanoenergetic coatings 80–100 microns thick. The dried layer was then ignited at one corner using a small flame-torch. The flame self-propagated extremely rapidly, and consumed the whole coated surface (~ 3 inch × 1 inch), after which it exhausted itself. The whole process (initiation, propagation, and quenching /exhaustion) took less than 1 second for the surfaces tested (1 inch × 3 inch pieces), and left behind a dark, flaky residue, which could be blown away and/or rinsed off to obtain the clean, biofilm-free surface underneath. Based on our earlier studies of the similar blends for other applications [21], the residue is believed to be carbonaceous, with minor amounts of aluminum oxide. (Most of the aluminum is oxidized to AlF_3_ by the fluoropolymer). The amount of acetone and the weight ratio of Al nanoparticles to THV 220A were optimized by observing how well the flame self-propagated upon ignition throughout the surface. More importantly, during this optimization, it was ensured that the swiftly propagated flame only destroyed the biofilm, while not significantly damaging the substrate underneath.

Upon combustion, nano-aluminum is known to produce temperatures of 2200 K–3200 K [18]. Such high temperatures are likely to destroy biofilms and organisms harbored within. Because the duration of the temperature pulse is small, (less than 10 ms at any point on the substrate) and the amount of heat imparted is relatively low (0.1 kJ/cm^2^), materials such as metals and ceramics that have high thermal diffusivity, are unaffected by the process.

### Assessment of bacterial numbers in biofilms (before and after combustion)

To estimate the surface density (number of bacteria per unit area) of bacteria present in the biofilm that grew on a coupon, we performed the following procedures: First, using an autoclaved razor, we shaved off the biofilm from the surface of interest. The shavings were collected in a plastic centrifuge tube with 7 ml of sterile phosphate buffered saline (1× PBS) solution. The surface and razor blade were also rinsed with PBS after shaving, and the rinsed solution pooled with the solution into which the shavings were deposited. The volume was then made up to 10 ml. In order to disperse the bacteria lodged in the peptidoglycan matrix, the sample was vortexed and then sonicated at low power in a Branson 2510 sonicator for 2 minutes. An estimate of the total number of bacteria present in this 10 ml volume was obtained by serial dilution and plate-counting. Briefly, this standard laboratory procedure [22] involved diluting the sample progressively over 9 orders of magnitude, then taking a 50 μl aliquot from each of them and spreading them over a petridish with Tryptic Soy Agar. The petridishes were incubated for ~48 hours, and the number of colonies was counted. Based on the colony counts from plates with 20–200 colonies, the number of colony forming units of bacteria present in the original 10 ml sample was estimated. The number of bacterial colony forming units present per unit surface area in our biofilm is obtained by dividing this number by the surface area of our coupon (biofilm).

In order to obtain the number of bacteria surviving our ultra-rapid combustion procedure, we collected the entire carbonaceous residue that we obtained at the end of the process, and dispersed it in 5 ml of PBS. We also scraped off the surface of interest, rinsed both the surface and the razor, and collected all the material together. As earlier, the volume was made up to 10 ml, the bacteria were dispersed using vortexing and sonication, and their number was estimated using serial dilution and plating. In many of the experiments (especially for the second and third sets of data that we obtained), we eschewed serial dilution during this part and obtained plate counts directly from the 10 ml of sample. Again, based on the number of colonies observed, an estimate of the number of viable bacteria present per unit area of the coupon (biofilm) surface was obtained.

## Results and discussion

### Estimates of the numbers of live bacteria in the biofilms grown

**Figure 2:**
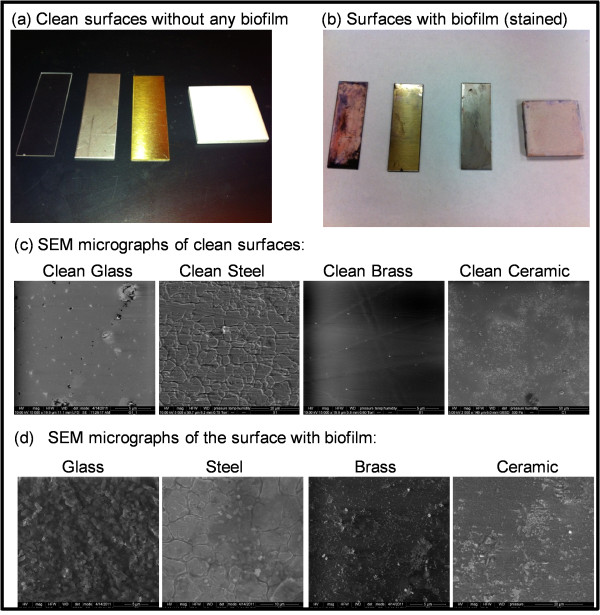
Images of substrates with and without biofilm and their corresponding SEM micrographs: (a) Photographs of the clean coupons before any biofilm was grown on it, (b) Photograph showing the coupons after the growth of biofilm on the surface with Gram staining and (c) SEM micrographs the clean coupons before the growth of biofilms (Left to Right: Glass, Steel, Brass and Ceramic) and (d) SEM micrographs of the coupons with biofilm (Left to Right: Glass, Steel, Brass and Ceramic).

The substrates, with and without biofilm, are shown in Figure 2. Also shown are scanning electron microscope (SEM) images of the biofilms. The number of viable bacteria per unit (nominal) area of these biofilms was obtained using the methods described in the previous section. These numbers are summarized in Table 2.

**Table 2:** Average, and standard deviation (n = 3) of the numbers of bacteria in the biofilms grown on various substrates

**Substrate**	**Bacterial load in biofilm (mean ± standard deviation)**
**Glass**	1.86 (± 0.47) × 10^7^
**Ceramic**	1.26 (± 0.46) × 10^8^
**Brass**	5.43 (± 0.23) × 10^7^
**Steel**	1.20 (± 0.34) × 10^8^

As seen, the numbers that we find are in the order of 10^7^ - 10^8^ CFU/cm^2^. The SEM images also show approximately 25 to 35 bacteria in rectangular regions whose lengths and widths are less than 20 microns each, yielding estimates of ~10^7^ - 10^8^ bacteria/cm^2^. These numbers also happen to be consistent with values reported elsewhere [2,11] for *P.aeruginosa* biofilms grown under moderate shear. Thus, our results also serve to verify that our method for growing biofilms is effective, and that our method for estimating bacterial surface densities is an acceptable one.

### The rapid combustion process

**Figure 3:**
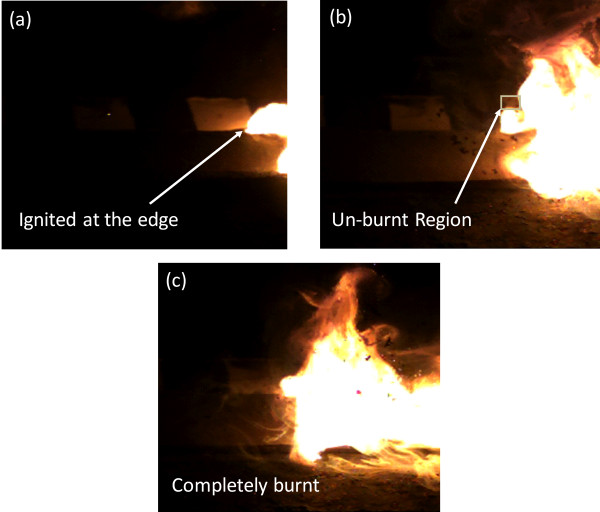
**Substrate subjected to the novel process of ultra-rapid biofilm removal.** The time arrival record of the flame front at different positions was determined using a high speed camera over a distance of 6 cm. (**a**) Initiation of the ignition at the edge of the coupon (time t = 0 ms) (**b**) The coupon with a small un-burnt region (time t = 30 ms) and (**c**) Surface of the coupon completely burnt (time t = 60 ms).

**Figure 4:**
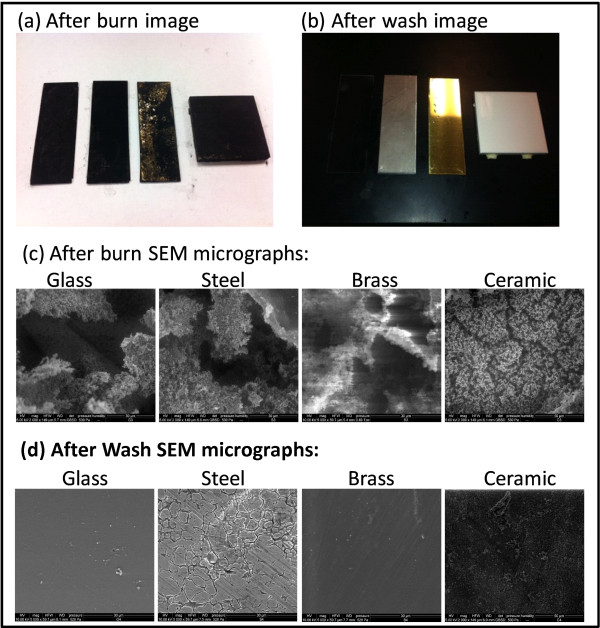
Images of substrates after burn and after wash with corresponding SEM micrographs: (a) Photograph of the coupons after the combustion process, showing the carbonaceous residue (b) Photograph showing the coupons after removal of the residue by washing (c) SEM Micrographs of the material surfaces after ultra-rapid biofilm combustion and (d) SEM micrographs of the coupons after removal of the carbonaceous residue by washing (Left to Right: Glass, Steel, Brass and Ceramic).

Pictures of the samples at various stages of the process are shown in Figure 3. (A video of the rapid combustion is also provided in the supplementary material). The first picture [Figure 3a] shows a coupon being ignited (after being spray-coated with the blend of fluoropolymer and aluminum nanoparticles dispersed in acetone, and allowing the acetone to subsequently evaporate). Subsequent images [Figure 3b and c] are taken 30 ms apart and as seen, the whole 3″ × 1″ top surface of the coupon burnt in < 100 ms. As recorded using a high speed camera, the flame propagated from the bottom-right to the top left corner, covering the diagonal length of the coupon (a distance of ~ 8 cm) in about 60 ms, thus yielding a linear propagation rate of about 1.3 m/s. As can been seen in Figure 4a, the combustion process left behind a carbonaceous residue, which was easily washed off from the surface to obtain the desired biofilm-free substrate (as shown in Figure 4b). Using SEM, we also examined the microstructure of the materials after it had been subjected to combustion (as shown in Figure 4c). On comparing the micrographs shown in Figure 4 to those in Figure 2, we were unable to observe any major damage or change to any of the materials.

### Estimates of the efficacy of the process

**Figure 5:**
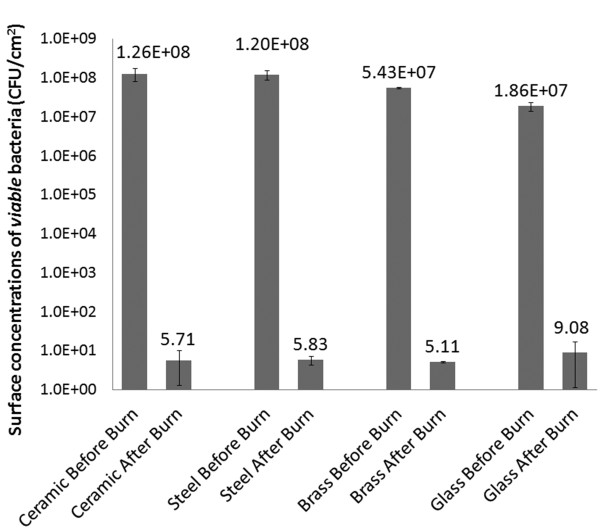
**Efficacy of our ultra-rapid process for the removal of biofilms: The graph shows the average (n = 4) and standard deviations for the surface concentration of viable bacteria (CFU/cm**^**2**^**) on different materials before and after the combustion process.**

The number of viable bacteria present on the surface of the substrates (coupons) after the combustion (including those on the carbonaceous material, if any) is estimated using the method described earlier. This number also includes any bacteria present on the carbonaceous material (if any). These numbers (divided by the nominal area of the coupon surface to obtain the numbers per unit area), are plotted in Figure 5 along with the numbers present per unit area in the biofilm prior to the rapid combustion process.

As seen in Figure 5, the method developed in the present work is able to reduce the numbers of bacteria on the surfaces to ~10 CFU/cm^2^. This represents a 5 to 6 log_10_ decrease from its original concentration. It may also be noted that the rapid combustion process, and the subsequent collection of carbonaceous material was performed in an environment that was *not* sterile, and hence random bacteria from the environment were likely to have been introduced into our “after” sample, leading to the estimate being higher than the true value of viable bacteria remaining.

In Table 1, we compared the efficiency of the rapid combustion process developed in this work to a few other methods reported in literature for eliminating bacteria / biofilms on solid substrates on two criteria: the degree to which the process is able to reduce the infestation of bacteria (as determined by the log-reduction in the number of viable bacteria), and the time taken to carry out the process. We compare our process of biofilm removal only to others that are used to treat process equipment or surfaces “offline”, and we do not foresee our combustion process being used to eliminate biofilms while a chemical or biological process is still ongoing. (The latter category includes biofilms on the surfaces of implanted medical devices such as orthopedic joints, pacemakers etc.)

As seen, the performance of our process is better that of the other methods listed on one or both counts. Firstly, its efficacy in eliminating viable bacteria harbored in biofilms is really high (we obtain a > 5 log reduction, as compared to 2–3 logs for most other processes), and secondly, its turn-around time is really short, with the core combustion process taking less than 1 second and the prior preparation steps (spraying) taking about 1 minute. Taken together, these two features make it an extremely promising commercial technology, especially for applications involving “offline” removal of biofilms from metallic and ceramic surfaces of process equipment, where both efficacy of removal and turn-around time are the key considerations. The other advantage of our method is that it generates very little solid or liquid wastes (the disposal of which typically requires additional resources to be expended).

While the experiments reported here demonstrate the potential of this approach, much work remains to be done before we can claim that it is ready for use in the real world. For instance, though we suspect that the carbonaceous residue that we obtained post-combustion may contain traces of the substrate material (metal or ceramic), quantification of these trace elements is beyond the scope of the current work. Depending on the amount of material lost, our process may be deemed acceptable for certain applications, and unacceptable for others. Also, we are not sure if any aerosols containing live bacteria are released during the process. If found to be so, additional precautions may be needed prior to using our method in real world situations. In addition, we will have to demonstrate that the process can successfully remove the older, more complex, mixed culture biofilms that are usually seen in process equipment, and investigate how the presence of chemical (as opposed to biological) fouling products (such as rust) affect the performance of this process. Additional issues that we will have to consider include formulating a blend that remains safe to handle even when used in large quantities and under challenging conditions, and formulating slower burning and/or less high-temperature blends for polymeric/plastic surfaces.

## Conclusions

The method of biofilm removal via the combustion of a nanoenergetic coating reported in this work is presently in an early stage of development. While much work remains before the method can be used in “real-world” settings, the present work nevertheless provides the scientific community with essential information regarding this novel method.

## Competing interests

The authors declare that no competing interests exist.

## Authors’ contributions

The nanoparticle-blend was formulated and prepared by RT and BMS, with guidance from KG and SG. The biofilm growth, ablation and evaluation were done by BDL, SP and BMS, with the guidance of SS. SS directed the overall study. All authors approve the final manuscript.
